# The risk of bleeding for antiplatelet agents in Haemodialysis patients: a Meta-analysis

**DOI:** 10.1186/s12882-020-01757-1

**Published:** 2020-03-26

**Authors:** Qi Wang, Xiaojie Xie, Gaosi Xu

**Affiliations:** 1 Department of Nephrology, the Second Affiliated Hospital of Nanchang University, No. 1, Minde Road, Donghu District, Nanchang, 330006 People’s Republic of China; 2 Department of Nephrology, 908 Hospital of People’s Liberation Army, Yingtan, China

**Keywords:** Haemodialysis, Anticoagulants, Aspirin, Clopidogrel, Bleeding

## Abstract

**Background:**

The safety of antiplatelet therapy in haemodialysis (HD) patients remains controversial. we conducted the first meta-analysis to evaluate the bleeding risk with antiplatelet agents in these populations.

**Methods:**

The relevant literature was searched using the following electronic databases without any language restrictions: the Cochrane Library, EMBASE, Global Health, MEDLINE, PubMed, and the Chinese Biomedical Database.

**Results:**

Seven randomized controlled trials (RCTs) and 2 prospective cohort studies, consisting of 1131 patients, were identified for detailed evaluation. The meta-analysis suggested that the use of double antiplatelet agents increased the risk of bleeding in HD patients [odds ratio **(**OR) = 2.78; 95% confidence interval (CI) 1.63 to 4.76; *I*^2^ = 0], and antiplatelet agents increased the risk of bleeding in 7 RCTs [odds ratio (RR) = 1.40, 95% CI 1.08 to 1.79; *I*^2^ = 23%,]; however, the use of a single antiplatelet agent was not found to significantly increase the risk of bleeding **(**RR = 0.88; 95% CI 0.51 to 1.50; *I*^**2**^ = 0).

**Conclusion:**

The results suggested that the use of double antiplatelet agents increased the risk of bleeding in HD patients**.**

## Background

More than 2 million people require maintenance haemodialysis (HD) globally, and this number is increasing by approximately 10% each year [1]. At least half of all patients starting dialysis therapy have overt cardiovascular disease (CVD) [2]. Antiplatelet agents, especially aspirin, have been used as primary and secondary prevention for CVD. Further, antiplatelet agents in haemodialysis patients are also used as a routine treatment to prevent ischaemic events after percutaneous intervention and are prescribed to prevent arteriovenous graft thrombosis [3]. HD patients are generally believed to have an increased risk of haemorrhage due to platelet dysfunction and altered platelet-vessel wall interactions, in addition to the factors that inhibit normal platelet adhesion and aggregation [4–6]. The risk of bleeding is especially high in HD patients because of the use of heparin during dialysis. Moreover, long-term therapy with aspirin is associated with a significant increase in the risk of haemorrhage [7].

Studies on antiplatelet agents evaluating the bleeding risk for HD patients have produced inconsistent results. Several cohort studies [8–10] showed that the risk of bleeding among antiplatelet agents remained unchanged in HD patients. In contrast, Eduardo et al. [11] found that antiplatelet agents were correlated with a significantly increased risk of bleeding. Although there has been no meta-analysis on the use of antiplatelet agent therapy for HD patients thus far, a systematic review by Hiremath et al. [12] suggested that the risks and benefits of antiplatelet agent treatment in HD patients remain poorly defined. In addition, the risk of bleeding in HD patients appears to be related to the number of antiplatelet agents used.

Therefore, we conducted the first meta-analysis to evaluate the bleeding risk of HD patients treated with antiplatelet agents, which specially aimed to explore the impact of the combined use of antiplatelet agents on bleeding risk in HD patients.

### Literature search

Relevant articles were collected using the following electronic databases: the Cochrane Library, EMBASE, Global Health, MEDLINE, PubMed, and the Chinese Biomedical Database from the building time of the database to July 2018. Keywords included: “anticoagulants” or “anticoagulation agents” or “anticoagulant agents” or “anticoagulant drugs” and “hemodialysis” or “hematodialysis” or “dialysis” or “dialyze” or “dialyse” or “dialys” and “bleeding” or “hemorrhage”. Abstracts, citation titles and the related research references were independently reviewed at the same time.

### Study criteria

The studies were included if they met the following criteria: (a) the study design should be a clinical cohort or prospective cohort that included thirty or more haemodialysis patients, (b) studies had three or more follow-up months to ensure that bleeding rates were related to antiplatelet exposure, and (c) studies also had to have assessed bleeding risk with antiplatelet agent treatment. The exclusion criteria were: (a) duplication, (b) studies of patients with peritoneal dialysis or who had recovered renal function and had transferred out of the dialysis programme, and (c) studies such as systemic reviews, meta-analyses, comments, retrospective studies, case reports, and animal experimental studies.

### Data extraction

**Fig. 1:**
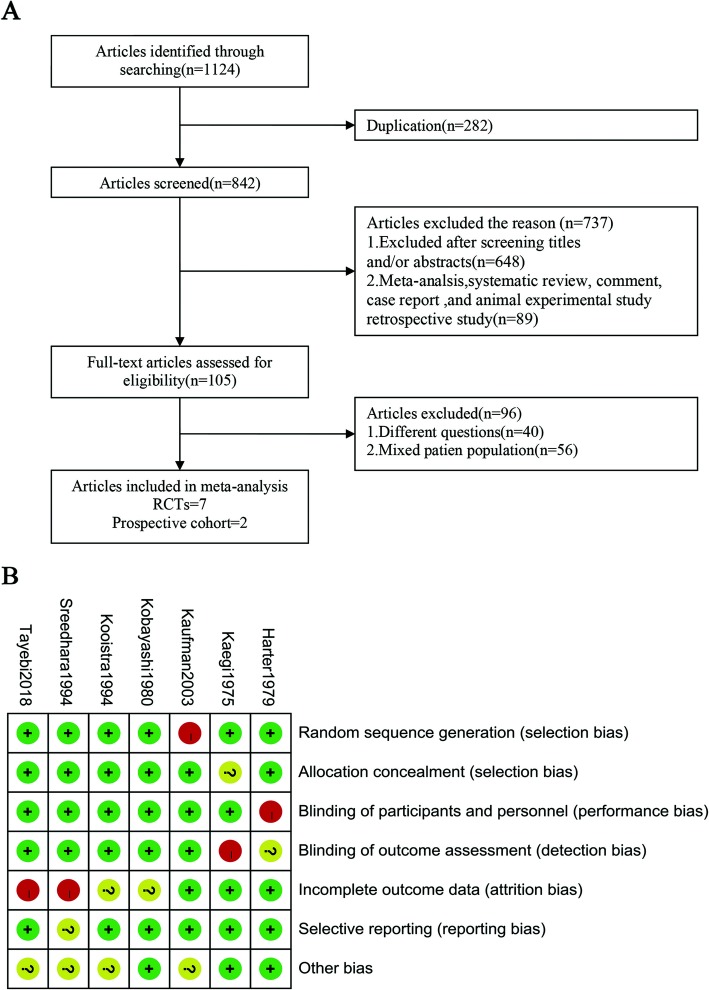
**a**. Flow diagram of the search results and selection of studies; **b**. Risk of bias summary

Two evaluators (QW and XX) independently extracted the data. We searched all potentially eligible citations to identify studies that met the criteria. Discrepancies were settled by a meeting consensus. Disagreements regarding the extracted data were solved through debate to reach a consensus. The details of the selection process are shown in Fig. 1a. Data extraction included the first author’s last name, year of publication, the control group, intervention measures, bleeding events (definition of bleeding events and number of bleeding events), number of patients, study duration and study quality.

### Assessment of study quality

A Review Manager (version 5.3) risk-of-bias assessment to evaluate study quality for the randomized controlled trials (RCTs) was conducted and included four sections: selection, performance/detection, attrition and reporting bias (Fig. 1b). The Newcastle–Ottawa quality assessment scale (range 0 to 9 stars) was used to evaluate the prospective study quality (Table 1). Stars were awarded for cohort studies after evaluation of selection, comparability and outcomes. A study could be given a maximum of two stars for comparability. No more than one star could be awarded for selection and exposure categories [22].

**Table 1:** Included studies of antiplatelet use in HD patients: Study design, treatments, intervention, bleeding events (A: Aspirin P: Placebo S: Sulfinpyrazone C: Control Cl: Clopidogrel A + Cl: Aspirin + Clopidogrel D: Dipyramidole A + D Aspirin + Dipyramidole)

Study	Year	Study design	Control group	Intervention	Bleeding		Bleeding events	total	Periods (months)	Quality score
					Defined a priori	Defined as used	Bleedings/patients (n/n)			
Harter [13]	1979	RCT	Placebo	Aspirin 160 mg od	No	Transfusions	A: 5/19 P: 13/25	44	5	M
Kaegi [14]	1975	RCT crossover	Placebo	Sulfinpyrazone 200 mg tid	No	GI bleedings	S: 2/45 P: 1/45	45	6	M
Kaufman [15]	2003	RCT	Placebo	Aspirin 325 mg + Clopidogrel 75 mg daily	Yes	As major, intermediate and minor	A + Cl:44/104 P: 23/96	200	~ 7	H
Kobayashi [16]	1980	RCT	Placebo	Ticlopidine 100 mg bid	No	Major or serious bleeding	T: 4/47 P: 3/53	100	3	H
Kooistra [17]	1994	RCT crossover	Placebo	Aspirin 30 mg od	No	Bleeding incidents not due to HD	A:2/137 P:5/137	137	3	M
Liu [18]	2016	Prospective cohort	Control	Aspirin 100 mg od	Yes	Intracranial hemorrhage, major bleeding events (i.e. gastrointestinal bleeding)	A: 14/152 C:36/254	406	60	6
Obialo [19]	2003	Prospective cohort	Control	Aspirin 325 mg od	No	GI bleedings	A: 5/21 C: 0/31	52	~ 4	7
Sreedhara [20](a)	1994	RCT	Placebo	Dipyramidole 75 mg tid	No	GI adverse events	D: 5/29 P: 2/24	107	72?	L
Sreedhara [20](b)			Placebo	Aspirin 325 mg daily			A: 3/26 P: 2/24	107		
Sreedhara [20](c)			Placebo	Aspirin 325 mg daily + Dipyramidole 75 mg tid			A + D: 5/28 P: 2/24	107		
Tayebi [21]	2018	RCT	Placebo	Aspirin 80 mg daily + Dipyramidole 75 mg daily	No	Major or serious bleeding	A + D: 2/20 P: 0/20	40	12	H

### Statistical analysis

The data were calculated by Review Manager (version 5.3) and STATA statistical software (version 12.0). Estimations of effect were summarized by forest plots, which data was expressed as risk ratios (RRs) with 95% confidence intervals (CIs) for dichotomous outcomes. Heterogeneity was estimated by using *Q* statistic and *I*^*2*^ tests (*I*^2^ > 50, 25% < *I*^*2*^ < 50%, and *I*^2^ < 25% represent high heterogeneity, moderate, and mild, respectively) [22]. A random-effects model was applied to process data with light heterogeneity in the results, whereas a fixed-effects model was adopted to process data in poor heterogeneity. In all statistical tests, a *P* ≤ 0.05 was used to indicate significance. We performed sensitivity and subgroup analyses to investigate the sources of heterogeneity. Publication bias and the differences in the studies were explored by using Begg’s and Egger’s funnel plots. Meta-regression was applied to test the variables such as the study design and the number of antiplatelet agents used. When zero occurrs in the counting data, in RevMan 5.3 software the automatic default is 0.5, which does not affect the results of RR and risk difference (RD) [23].

## Results

### Characteristics and quality of the included studies

Nine clinical trials [13–21] with 1131 patients ultimately met the inclusion criteria (Fig. 1a). These included 7 RCTs [13–17, 20, 21] and 2 prospective cohort studies [18, 19]. Two of the RCTs had a crossover design, and one of them (Kaegi) occurred the bleeding events only in the initial phase before crossover. The other study (Kooistra) had a one-week washout period. Single antiplatelet agents (including aspirin, ticlopidine, clopidogrel, sulfinpyrazone or dipyridamole) were used in 7 studies [13, 14, 16–20]. Double antiplatelet agents were used as an intervention group in 3 studies [15, 20, 21]. The methodological quality and characteristics of all the included studies are shown in Table 1.

### Antiplatelet use with the bleeding risk

**Fig. 2:**
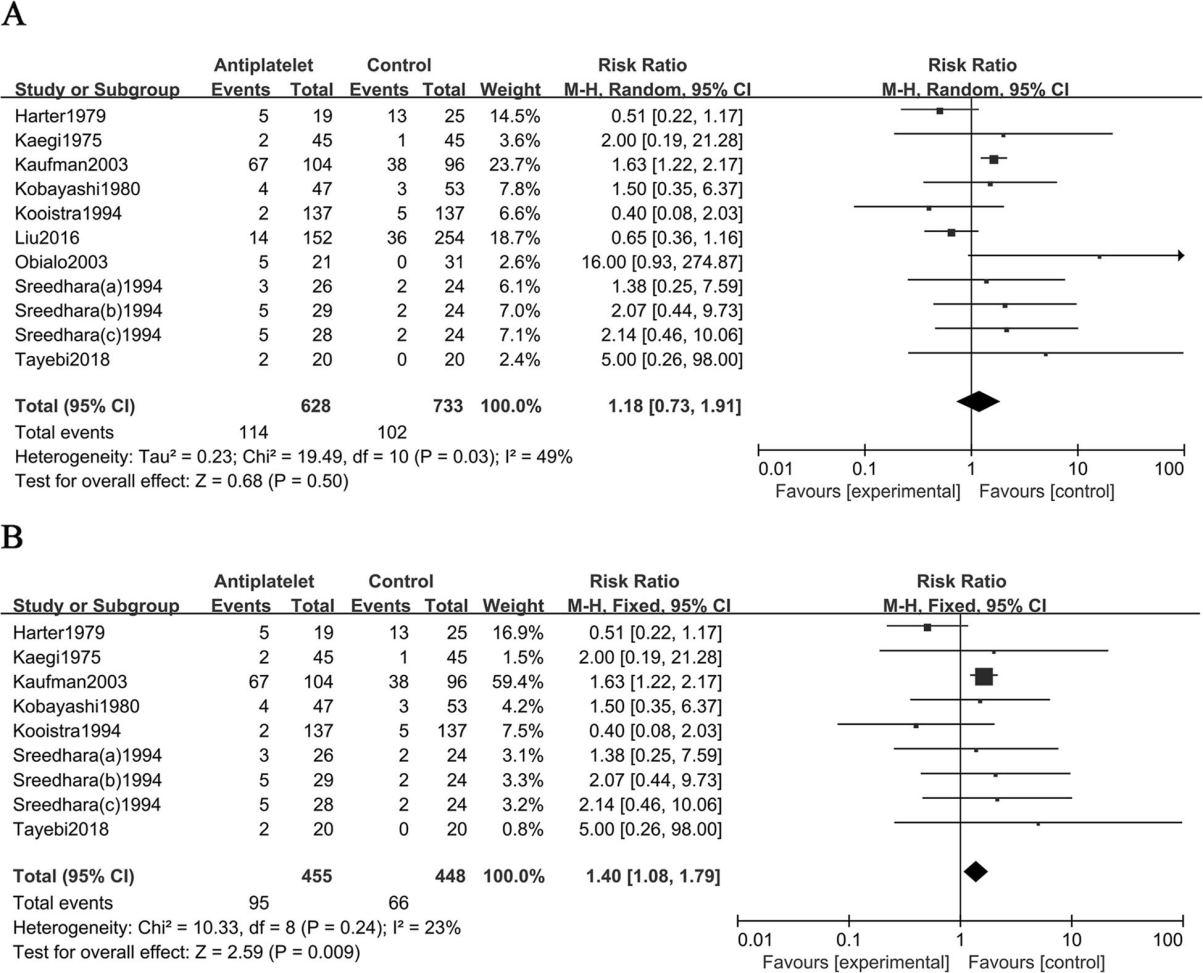
**a**. Forest plots of all 9 studies showing the bleeding risk of antiplatelet agent use in HD patients; **b**. Forest plots of 7 RCTs showing the bleeding risk of antiplatelet agent use in HD patients

A total of 628 out of 1131participants in 9 studies received antiplatelet agents. There was statistical heterogeneity between the studies (*I*^2^ = 49%, *P* = 0.03, Fig. 2a**)**; thus, a random-effects model was selected. The results indicated that the use of antiplatelet drugs and the risk of bleeding were not statistically significant (RR = 1.18, 95% CI 0.73 to 1.91; *P* = 0.50). A total of 455 out of 673 participants in 7 RCTs [13–17, 20, 21] received antiplatelet agents. There was no statistical heterogeneity between the included studies (***I***^**2**^ = 23%, *P* = 0.24, Fig. 2b); thus, a fixed-effects model was selected. The results indicated that the use of antiplatelet agents increased the risk of bleeding (RR = 1.40, 95% CI 1.08 to 1.79; *P* = 0.009). Subgroup analysis was necessary to clarify the source of the high heterogeneity that we identified and to increase the reliability of the results.

### Subgroup analysis of antiplatelet agents

**Fig. 3:**
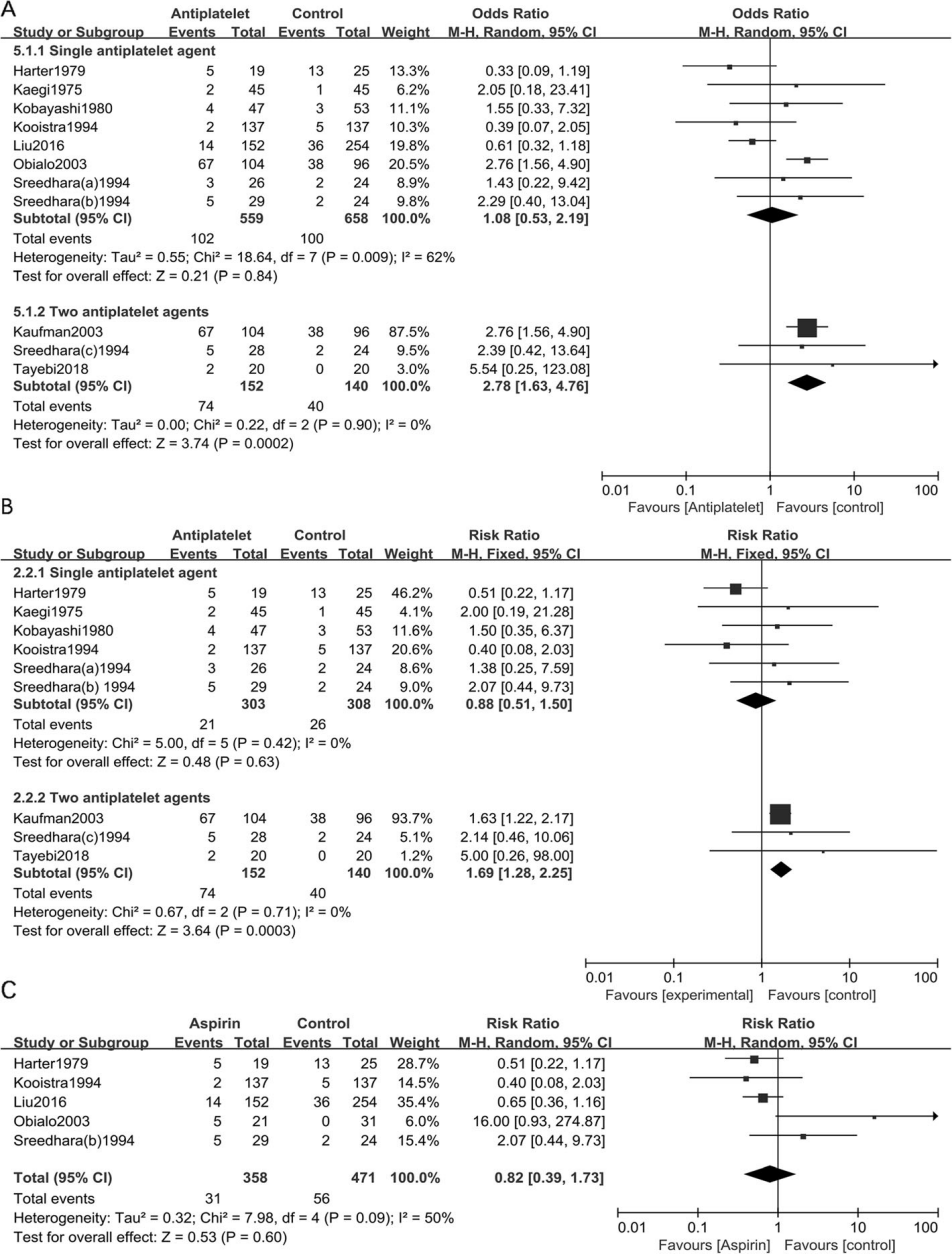
**a**. Subgroup analysis of the use of a single antiplatelet agent or two antiplatelet agents and the risk of stroke in haemodialysis patients in all 9 studies; **b**. Subgroup analysis of the use of a single antiplatelet agent or two antiplatelet agents and the risk of bleeding in haemodialysis patients in 7 RCTs; **c**. Subgroup analysis of aspirin monotherapy and the risk of bleeding in haemodialysis patients

**Fig. 4:**
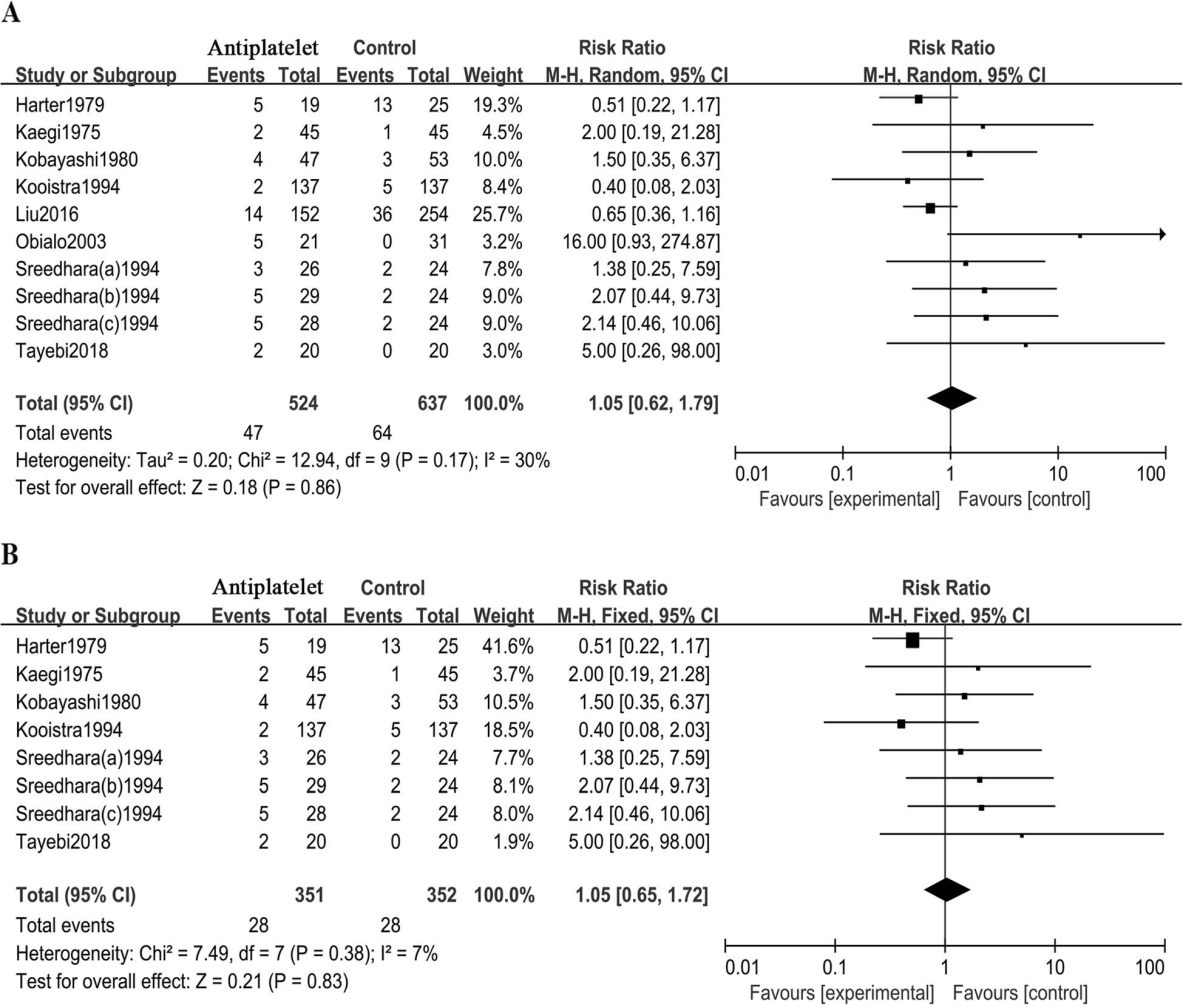
**a**. Subgroup analysis of the use of antiplatelet agents and the risk of bleeding in haemodialysis patients when all studies other than Kauffman 2003 were considered; **b**. Subgroup analysis of the use of antiplatelet agents and the risk of bleeding in haemodialysis patients in 6 RCTs when Kaufman 2003 was excluded

A subgroup analysis based on the number of antiplatelet agents (Fig. 3a) found that the use of double antiplatelet agents increased the risk of bleeding in HD patients (RR = 2.78; 95% CI 1.63 to 4.76; *I*^**2**^ = 0), while use of a single antiplatelet agent was not significantly associated with the risk of increased bleeding [RR = 1.08; 95% CI 0.53 to 2.19; *I*^***2***^ = 62]. Another subgroup analysis based on the number of antiplatelet agents in 7 RCTs (Fig. 3b) demonstrated that the use of double antiplatelet agents increased the risk of bleeding in HD patients (RR = 1.69; 95% CI 1.28 to 2.25; *I*^***2***^ = 0), while the use of a single antiplatelet agent and the risk of bleeding were not statistically significant (RR = 0.88; 95% CI 0.51 to 1.50; *I*^**2**^ = 0). An additional subgroup meta-analysis of aspirin monotherapy vs placebo (Fig. 3c) found that aspirin monotherapy and the risk of bleeding was not statistically significant (RR = 0.82; 95% CI 0.39 to 1.37; *I*^*2*^ = 50). Moreover, when all studies other than Kauffman 2003 were considered, antiplatelet agents and the risk of bleeding were still not statistically significant (RR = 1.05; 95% CI 0.62 to 1.79; *I*^*2*^ = 30, Fig. 4a). Additionally, antiplatelet agents and the risk of bleeding were not statistically significant (RR = 1.05; 95% CI 0.65 to 1.72; *I*^*2*^ = 7, Fig. 4b) in 6 RCTs when Kaufman 2003 was excluded.

### Sensitivity analyses and publication bias

**Fig. 5:**
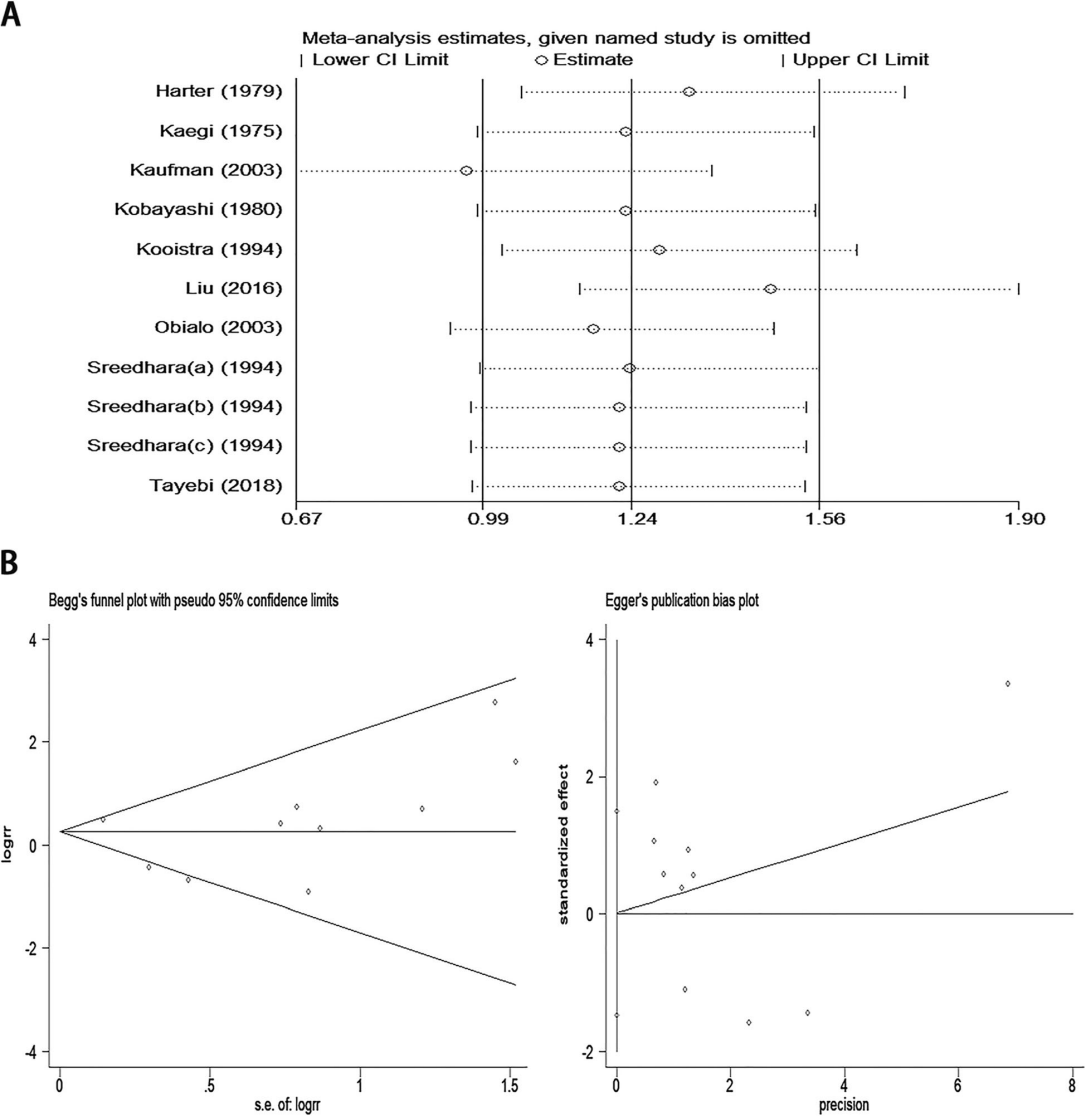
**a**. Sensitivity analyses of the bleeding risk of antiplatelet agent use in haemodialysis patients; **b**. Publication bias according to Egger’s and Begg’s test funnel plots.

Sensitivity analyses were carried out considering the bleeding risk in haemodialysis patients. There were significant effects on the results of the HR and 95% CI when the Kaufman et al. [15] study was excluded (Fig. 5a), indicating that this study had high sensitivity and poor stability. To detect publication bias, Egger’s and Begg’s test funnel plots were used (Fig. 5b). There was no evidence of substantial publication bias according to Egger’s linear regression test (*P* > 0.987) and Begg’s rank correlation test (*Pr* > |z| = 0.243) in this meta-analysis.

### Sources of heterogeneity and meta-regression

**Fig. 6:**
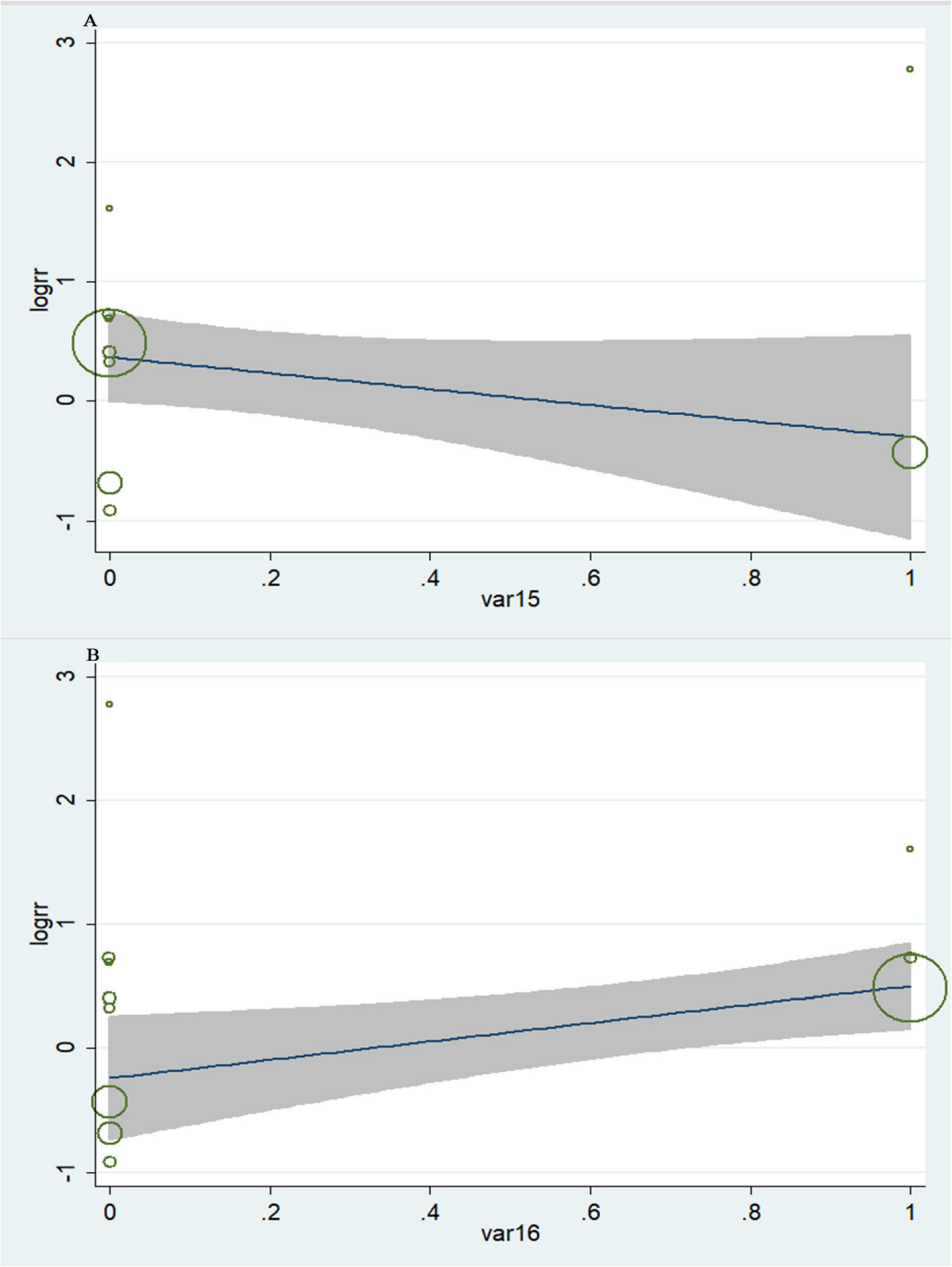
**a**. Meta-regression based on study design about the association between antiplatelet use and the risk of bleeding; **b**. Meta-regression based on the number of antiplatelet agents used and the association between antiplatelet use and the risk of bleeding

Meta-regression was performed to explore the sources of heterogeneity. The relationship between antiplatelet agents and the risk of bleeding was not influenced by study design (*P* = 0.654, Fig. 6a). However, the number of antiplatelet agents used showed impact on the bleeding risk in HD patients (*P* = 0.021, Fig. 6b).

## Discussion

The type of antiplatelet agen and the prescribed number of antiplatelet agents appear to be related to the bleeding risk for HD patients [12]. In HD patients, antiplatelet agents may lead to more bleeding events because of platelet dysfunction and differences in haemodynamic stability [24]. More importantly,the standard of care for patients with acute coronary syndrome and undergoing percutaneous coronary intervention is the combination of two antiplatelet agents, partially because of the results of the CURE study [25, 26]. This meta-analysis included 9 articles involving 1131 hemodialysis patients, we found that HD patients have an increased risk of bleeding due to the use of double antiplatelet agents However, while the point estimate for the studies (aspirin + dipyramidole) suggests risk, they contributed only 12.5% of the weight to the meta-analysis. Thus, aspirin + clopidogrel confers a bleeding risk, and aspirin + dipyramidole might also confer a bleeding risk. Furthermore, use of a single antiplatelet agent did not appear to be associated with bleeding risk in the subgroup analysis.

To explore the sources of the heterogeneity, we performed the subgroup analysis based on the study design and the number of antiplatelet agents. The former was not statistically significant, but the latter was statistically significant. Second, Kaufman et al. [15] might be one of the main sources of heterogeneity. Because Kaufman et al. [15] had the best scientific rigor, we posit that the source of heterogeneity was dosage and the combination of antiplatelet agents. Egger’s and Begg’s test funnel plots showed no publication bias in this meta-analysis. Finally, the meta-regression included two variables that study design and number of platelet agents. Study design was not heterogenous, but the number of platelet agents could affect the heterogeneity. Moreover, regarding the high heterogeneity, the type of antiplatelet agent might affect the heterogeneity.

There were several limitations to our study. First, the classes of antiplatelet agents and intensities of platelet inhibition were different. Second, the definition of bleeding complications was not consistent in the included studies, which might lead to the different bleeding risk in HD patients. Third, there was lack of large randomized clinical trials in our research, which should be further explored and confirmed. Fourth, patients on dialysis treated with heparin may have increased the bleeding risk.

## Conclusion

In conclusion, we performed the first meta-analysis assessing the bleeding risk associated with the use of antiplatelet agents in HD patients. The results suggested that double antiplatelet agents should not be recommended for routine treatment in HD patients, especially the combination of aspirin + clopidogrel. In contrast, single antiplatelet agents were not found to significantly increase the risk of bleeding.

## Data Availability

All data generated and analysed during this study are included in this published article.

## Abbreviations

HD: Haemodialysis
RCTs: Randomized controlled trials
OR: Odds ratio
RR: Risk ratio
CI: Confidence interval
CVD: Cardiovascular disease
RD: Risk difference
